# Workplace Mental Health Disclosure, Sustainable Employability and Well-Being at Work: A Cross-Sectional Study Among Military Personnel with Mental Illness

**DOI:** 10.1007/s10926-022-10083-2

**Published:** 2022-11-14

**Authors:** Rebecca Bogaers, Elbert Geuze, Jaap van Weeghel, Fenna Leijten, Dike van de Mheen, Nicolas Rüsch, Andrea Rozema, Evelien Brouwers

**Affiliations:** 1 Tranzo, Scientific Center for Care and Well-Being, Tilburg School of Social and Behavioral Sciences, Tilburg University, Warandelaan 2, 5037 AB Tilburg, The Netherlands; 2 Brain Research and Innovation Centre, Ministry of Defence, Lundlaan 1, 3584 CX Utrecht, The Netherlands; 3 Strategic Military Healthcare Department, Ministry of Defence, Herculeslaan 1, 3584 AB Utrecht, The Netherlands; 4 Department of Psychiatry, Brain Center Rudolf Magnus, University Medical Center Utrecht, Heidelberglaan 100, 3584 CX Utrecht, The Netherlands; 5 Directorate of Strategy and Knowledge, Directorate-General of Policy, Ministry of Defence, Kalvermarkt 32, 2511 CB The Hague, The Netherlands; 6 Department of Psychiatry II, University of Ulm and BKH, Günzburg, Germany

**Keywords:** Well-being, Sustainable employability, Disclosure, Mental health, Stigma, Military

## Abstract

*Purpose* Disclosure of mental illness to a supervisor can have positive (e.g. supervisor support) and negative consequences (e.g. stigma). However, research on the association between disclosure and sustainable employability and well-being at work is scarce. The aim of this study was to investigate the association between the disclosure decision (yes/no), experiences with the decision (positive/negative) and sustainable employment and well-being at work among military personnel with mental illness (N = 323). *Methods* A cross-sectional questionnaire study was conducted. Descriptive and regression (linear and ordinal) analyses were performed. Comparisons were made between those with positive and negative disclosure experiences. *Results* Disclosure decision (yes/no) was not significantly associated with any of the measures of sustainable employability and well-being at work. However, positive disclosure experiences were significantly associated with higher scores on almost all measures of sustainable employability and well-being at work. Those with negative disclosure experiences reported significantly more shame (M_pos_ = 2.42, M_neg_ = 2.78, p < .05) and discrimination (M_pos_ = 1.70, M_neg_ = 2.84, p < .001). Those with a positive disclosure experience, reported significantly more supervisor support (M_pos_ = 3.20, M_neg_ = 1.94, p < .001). *Conclusion* We did not find evidence that the disclosure decision itself is related to measures of sustainable employment and well-being at work. In contrast, how participants had experienced their (non-)disclosure decision was significantly related to almost all measures. This emphasizes the importance of the work environments reactions to disclosure and mental illness in the workplace. Future research and interventions should focus on increasing the likelihood of positive disclosure experiences through creating a more inclusive work environment, with more supervisor support and less stigma.

**Supplementary Information:**

The online version contains supplementary material available at 10.1007/s10926-022-10083-2.

## Introduction

The importance of well-being at work for sustainable employability and mental health has increasingly gained attention [1, 2]. This is likely to increase even further in the coming decades due to the aging working population in Western countries [3], the current labor shortage in many industries [4], more importance given to work-life balance and flexibility [5] and workers searching increased compensation [6]. Additionally, the pandemic from covid-19 has impacted well-being at work and mental health [7]. Together this makes the topic of how employers can invest in workers’ health and well-being, a relevant topic.

Sustainable employability refers to the ability of workers to participate in work and the labor market during their lifetimes [8]. Traditionally, conceptualization of sustainable employability was mainly based on a medical and performance perspective, focusing on complaints (e.g. sick-leave [9]) and performance related indicators (e.g. work ability [10]). However, with the emerging subfield of positive organizational psychology [11], there is a shift in focus of conceptualization of sustainable employability, with more focus on well-being at work [8]. For instance, based on the capability approach, sustainable employability is defined as ‘throughout their working lives, workers can achieve tangible opportunities in the form of a set of capabilities. They also enjoy the necessary conditions that allow them to make a valuable contribution through their work, now and in the future, while safeguarding their health and welfare. This requires, on the one hand, a work context that facilitates this for them and on the other, the attitude and motivation to exploit these opportunities’ [8]. According to this approach, these conditions lead to well-being at work. As the conceptualization of sustainable employability has shifted to more focus on well-being at work, the current study will focus both on sustainable employability from a traditional perspective and on well-being at work by including more novel measures.

Colleagues and supervisors (e.g. the work environment) play a crucial role in well-being at work, and thus sustainable employability of workers [12, 13]. This is even more important for workers with mental illness (MI), including substance abuse, as illness is a threat to sustainable employability itself [14]*.* Workers with MI are amongst those with the highest risk of sick leave, disability pension and job loss, and recent studies increasingly suggest that this is not merely a result of their health problem, but also of workplace factors, such as a lack of supervisor and coworker support, stigma and discrimination [15–18]. As MI is common, with the lifetime prevalence of 29% in the global population [19], it is important to examine the ways in which workers with MI can be supported through the work environment.

A crucial decision for workers with MI, which affects the way the work environment responds to them, is the decision to disclose MI to a supervisor or not. Disclosure has been believed to positively impact sustainable employability and well-being through receiving workplace support and work accommodations, and non-disclosure can lead to a missed opportunity for this support [20–22]. However, a longitudinal study among unemployed people showed that those who were more reluctant to disclose their MI to (potential new) employers, were more likely to have found a job after six months [23]. Additionally, disclosure to a supervisor can lead to stigma and discrimination [24–26]. For example, of those who reported a negative disclosure experience in a study among Dutch workers, a quarter reported that they had been treated differently due to their disclosure [27]. In sum, disclosure decisions can both lead to positive and negative outcomes. The question whether the (non-)disclosure decision and disclosure experiences are positive or negative for sustainable employability and well-being at work is important, yet under researched.

The direct association between mental health disclosure to a supervisor and sustainable employability and well-being at work has, to the knowledge of the authors, not been examined in one study before. Most models on mental health disclosure end at the disclosure decision. They do not include consequences of disclosure, such as sustainable employability [28, 29], or do not focus on disclosure in the workplace [30]. A more complete model of disclosure, which does include consequences of disclosure in the work-context, is the *mental health condition decision-making process from antecedents to outcomes* model [31]. This model proposes that the short-term outcomes of disclosure are alleviation of inhibition, social-support, and changes in social information. The long-term outcomes include empowerment, individual outcomes (e.g. career development), dyadic outcomes (e.g. trust) and social contextual outcomes (e.g. experience of stigma). However, this model does not include the direct association between disclosure and sustainable employability and well-being at work, only the indirect association. Additionally, it should be noted that the data supporting this model is of qualitative nature. Associations should also be tested with quantitative data to further examine associations between disclosure and sustainable employability and well-being at work.

It is plausible that the decision to disclose or not is an even more prominent dilemma in high-risk occupations, such as the military. People in military professions are expected to be strong, and not to show weakness, possibly making disclosure harder [21, 24, 32]. For example, in a study in the German military, a soldier indicated “If I hear that this battalion commander is mentally ill … as a subordinate, honestly, I’d say ‘What kind of guy is that?’ … He must be a warrior, he must not be soft.” [33]. Additionally, previous research on disclosure in the military has also shown that military personnel fear that disclosure will lead to them not being considered fit for high-risk occupations, and there is a fear of negative career consequences such as loss of employment, not being able to advance in your career, or not being able to what you like best about your work [24]. Also, research suggests that disclosure might lead to more negative consequences in these types of occupations [22]. This makes it even more important to study disclosure and consequences for sustainable employability and well-being within high-risk occupations. Additionally, research on disclosure in the military is scarce [24, 33], and previous research has mainly focused on treatment seeking in the military and not on disclosure to a supervisor [32].

The current study aims to gain insight the association between MI (non-)disclosure decisions and experiences and sustainable employability and well-being at work. Specifically, the research questions of the current study were (1) ‘What is the association between disclosure decision (disclosure vs. non-disclosure) and sustainable employability and well-being at work?’, (2) ‘What is the association between disclosure experiences (positive vs. negative) and sustainable employability and well-being at work?,’ and (3) ‘What experiences do military personnel have concerning the (non-)disclosure decision (positive vs. negative), and what factors explain these experiences (how do those with a positive experience differ from those with a negative experience)?’.

## Method

### Design

A cross-sectional observational design with an online questionnaire was used. The questionnaire also contained more topics for other studies, e.g. treatment seeking for MI [34] and disclosure of MI to a supervisor (manuscript submitted for publication). The STROBE-checklist was used to report this study [35]. The study took place within the Dutch military.

### Participant Recruitment

Data were collected between January and February of 2021. Active-duty military personnel who had been on deployment were recruited for this study. For the larger questionnaire study including more topics, it was important that both personnel with and without mental illness would be represented in the sample. To ensure this, existing data from the questionnaire that personnel had received after deployment were used. Those who had been on deployment for 30 days or longer received this questionnaire 6 months after their deployment. It includes scores on depression, aggression, alcohol abuse, and PTSD. A stratified sample, based on gender, age, military division, and rank, of personnel was approached (N = 1000 with indication of mental illness and N = 1000 without). At the start of the questionnaire, personnel were asked whether they have (had) MI (self-reported). Only personnel who indicated having (had) MI were included in the current study (N = 323). All personnel were invited at the same time, both by e-mail and a letter. Reminders were sent after 3 and 5 weeks. It was made clear that the responses to the questionnaire would be anonymous.

### Measures

#### Demographics and Work Variables

Gender, age, marital status, education-level, type of work (combat units vs. combat support units) military department, rank, and years of service were assessed. Additionally, work demands were assessed, using a single item (*I think my work pressure is…*) on a 5-point scale, ranging from ‘way too low’ to ‘way too high’. This item was taken from the Questionnaire on the Experience and Evaluation of Work (QEEW) [36].

#### Mental Illness

##### Current MI

To assess current MI, the following measures were used; (a) Hospital Anxiety and Depression Scale [37], (b) ASSIST-LITE for substance abuse [38], (c) AUDIT-C, for alcohol abuse [39], and (d) PTSD checklist for DSM-5 [40]. For psychometric properties and cut-off scores, see Online Appendix A. The results of these questionnaires were only used for background information, not for the inclusion of participants in the current study.

##### Self-reported MI

Personnel were asked whether they have (had) MI. Only those who reported having (had) MI were included in the current study. If personnel reported having (had) MI, they received a list of 15 possible types of MI (see Online Appendix B) and were asked to indicate whether it concerned current or past MI, in line with earlier research [17, 34, 41]. In case the MI was in the past, they were asked the number of months since the MI ended. Additionally, they were asked whether the MI was work-related (yes/no) and to rate the severity of their symptoms (during the worst time) on a scale of 0 –10.

#### (Non-)Disclosure Decisions, General Experiences and Specific Experiences

##### Actual Disclosure Decision

Personnel were asked whether they had disclosed their MI to their supervisor themselves (yes/no).

##### General Disclosure Experience

Personnel were asked what their general experience was with their (non)-disclosure decision (very negative/negative/positive/very positive). No neutral response was provided and for the analyses very negative and negative were combined, and positive and very positive were combined. This was done to be able to make a comparison between those with a negative and positive experience.

##### Specific Disclosure Experiences

Following the question about the general experience, participants received statements about specific experiences and were asked to indicate on a 4-point scale whether they completely disagreed—completely agreed. An overview of the statements can be found in Table 1. Those who had disclosed received 15 statements (e.g. “*I felt supported by my supervisor*’ and ‘*I would have preferred to solve my own problems*’). Those who had not disclosed received 12 statements (e.g. ‘*Because I did not disclose, there were no negative consequences for my career*’ and ‘*I am glad my MI remained private*’). Statements were developed based on earlier research into disclosure experiences and included positive and negative factors contributing to the general experience [24, 27, 28, 41, 42]. All the participants that disclosed, received the same statements, and all the participants that did not disclose received the same statements. This means that those with a positive general experience and those with a negative general experience, both received statements about positive as well as negative factors contributing to the general experience. This was done, as positive and negative specific experiences can occur at the same time. Presence of a certain specific experience was assessed by combining ‘agree’ and ‘completely agree’.

**Table 1:** Disclosure decisions and experiences

Chose to disclose						
	Positive about disclosure decision N = 213, 86.9%		Negative about disclosure decision N = 32, 13.1%		Total N = 245	Difference
	N (%)	M (SD)	N (%)	M (SD)	N (%)	Significance
Positive factors contributing to experience						
Received support supervisor	192 (90.1)	3.20 (.66)	7 (21.9)	1.94 (.72)	199 (81.2)	**
Supervisor took mental illness seriously	197 (92.5)	3.24 (.63)	11 (34.4)	2.13 (.75)	208 (84.9)	**
Mental illness remained confidential	208 (97.7)	3.30 (.51)	16 (50.0)	2.44 (.62)	224 (91.4)	**
Disclosure improved relationships at work	124 (58.2)	2.62 (.70)	7 (21.9)	2.06 (.72)	131 (53.5)	**
Disclosure led to work adjustments	123 (57.7)	2.58 (.81)	8 (25.0)	2.09 (.73)	131 (53.5)	*
Could be authentic self due to disclosure	127 (59.6)	2.62 (.70)	6 (18.8)	2.03 (.60)	133 (54.3)	**
Could be example to others with MI due to disclosure	132 (62.0)	2.61 (.74)	10 (31.3)	2.19 (.64)	142 (58.0)	*
Disclosure was my own choice	197 (92.5)	3.25 (.66)	21 (65.6)	2.81 (.78)	218 (89.0)	*
Negative factors contributing to experience						
Felt shame about mental illness	107 (50.2)	2.42 (.85)	23 (71.9)	2.78 (.83)	130 (53.1)	*
Received blame for mental illness	19 (8.7)	1.73 (.63)	9 (28.1)	2.16 (.72)	28 (11.4)	*
There was gossip about me	63 (29.6)	2.07 (.77)	26 (81.3)	3.03 (.74)	89 (36.3)	**
Negative career consequences	15 (7.0)	1.69 (.63)	21 (65.6)	2.56 (.76)	36 (14.7)	**
Discrimination (treated differently by others)	17 (8.0)	1.70 (.64)	25 (78.1)	2.84 (.72)	42 (17.1)	**
Social rejection (perceived more negatively by others)	21 (9.9)	1.75 (.65)	19 (59.4)	2.53 (.80)	40 (16.3)	**
Would have preferred to solve my own problems (self-management)	108 (50.7)	2.40 (.87)	27 (84.4)	3.09 (.64)	135 (55.1)	**

Chose not to disclose						
	Positive about non-disclosure decision N = 66, 84.6%		Negative about non-disclosure decision N = 12, 15.4%		Total N = 78	Difference
	N (%)	M (SD)	N (%)	M (SD)	N (%)	Significance
Positive factors contributing to experience						
Preference to solve one’s own problems instead of disclosing	59 (89.4)	3.15 (.64)	9 (75.0)	3.0 (.74)	68 (87.2)	n.s
* If I had disclosed*, I would have felt embarrassed	29 (43.9)	2.35 (.89)	8 (66.7)	2.67 (.99)	37 (47.4)	n.s
* Because I did not disclose*, I experienced no discrimination	48 (72.7)	2.79 (.73)	6 (50.0)	2.58 (.90)	54 (69.2)	n.s
* Because I did not disclose*, there was no negative effect on career	34 (51.5)	2.44 (.84)	7 (58.3)	2.67 (.89)	41 (52.6)	n.s
* Because I did not disclose*, I experienced no social rejection	38 (57.6)	2.52 (.83)	5 (41.7)	2.50 (.91)	43 (55.1)	n.s
* Because I did not disclose*, I experienced no gossip	40 (60.6)	2.50 (.85)	4 (33.3)	2.33 (.78)	44 (56.4)	n.s
Mental illness did not influence occupational functioning	48 (72.7)	2.76 (.82)	7 (58.3)	2.58 (1.0)	55 (70.5)	n.s
* Because I did not disclose*, my mental illness remained private	53 (80.3)	3.36 (.87)	11 (91.7)	3.25 (.62)	64 (82.1)	n.s
Negative factors contributing to experience						
* Because I did not disclose*, I could not be my authentic self	20 (30.3)	2.14 (.80)	7 (58.3)	2.50 (.91)	27 (34.6)	n.s
* Because I did not disclose*, I missed the opportunity for work adjustments	14 (21.2)	1.95 (.85)	4 (33.3)	2.08 (1.0)	18 (23.1)	n.s
* Because I did not disclose*, I missed the opportunity for support supervisor	32 (48.5)	2.45 (.90)	9 (75.0)	2.67 (.65)	41 (52.6)	n.s
* Because I did not disclose*, I missed the opportunity to be example	28 (42.4)	2.35 (.83)	5 (41.7)	(.79)	33 (42.3)	n.s

^a^All the participants who disclosed, received the same statements, and all the participants who did not disclose received the same statements. Presence of a certain specific experience was assessed by combining ‘agree’ and ‘completely agree’

^b^n.s. = non-significant, *p < .05, ** p < .001

#### Measures of Sustainable Employability and Well-Being at Work

For the current study, several measures were used for sustainable employability and well-being at work, combining more traditional measures with more novel measures.

##### Turn-Over Intention

Turn-over intention was measured using 3 items from the QEEW [36] (e.g. ‘*I am planning to switch jobs in the coming year*’). The items were on a 5-point scale, ranging from ‘completely disagree’ to ‘completely agree’. The mean score was used for further analyses.

##### Work Ability

To assess work ability, a single item from the work ability index (WAI) was used. For this item, a person is asked to rate their current work ability compared with the life-time best, with a possible score of 0 (‘completely unable to work’ to 10 ‘work ability at its best’) [43].

##### Burn-Out

Burn-out was measured using the UBOS [44], a 15 item measure on a 7-point scale ranging from ‘never’ to ‘always’. The UBOS includes the subscales ‘emotional exhaustion’, ‘depersonalization’ and ‘personal accomplishment’. The items related to personal accomplishment were recoded. A mean score of the subscales was used for further analyses.

##### Job Satisfaction

Job satisfaction was measured with a single item ‘*In general, how satisfied are you with your job*’ on a 5-point scale ranging from ‘very unsatisfied’ to ‘very satisfied’, following earlier research [45].

##### Authenticity

Authenticity, the degree to which a person acts in agreement with one’s true self, was measured using the self-alienation sub-scale from the full I AM WORK scale [46]. For example, one of the items was ‘At work, I feel alienated’. Authenticity is important for sustainable employability and well-being at work [46]. The subscale consists of 4 items on a 7-point scale ranging from ‘completely does not apply to me’ to ‘completely applies to me’. Items were recoded such that a higher score, indicates more authenticity. The mean was used for further analyses.

##### Capability Set for Work

The capability set for work is a more novel measure of sustainable employability [2, 8, 9]. It measures a person’s values, whether they are given the opportunity by their work environment to realize this value, and whether they can personally manage to realize the value. The values are part of a person’s capability set when the participant finds the value important, they are given the opportunity to realize it, and they can personally manage to realize it. In that case, the value is given a score 1. The sum score of all the values was used as an indication for the capability set for work, with a minimum of 0 and a maximum of 7. Research shows this questionnaire is a valid measure for assessing a worker’s sustainable employability, with higher scores relating to higher sustainable employability [9].

### Statistical Analyses

All analyses were performed using SPSS. To test the association between disclosure decision and the measures of sustainable employability and well-being at work (research question 1), separate hierarchical linear regressions were performed per measure. The following variables were entered as the independent variables in step 1 of the hierarchical linear regression: demographics (gender, age, education), work context (combat unit vs. combat support unit, work demands), health variables (current vs. past MI, symptom severity) and disclosure decision (disclosure vs. non-disclosure). For job satisfaction an ordinal logistic regression was performed, as job satisfaction was not normally distributed. Job satisfaction included 5 categories ranging from ‘very unsatisfied’ to ‘very satisfied’.

To test the association between disclosure experience (positive vs. negative) and the measures of sustainable employability and well-being at work (research question 2), the same hierarchical linear regression analyses were used, but now disclosure experience was entered as an independent variable in step 2 of the hierarchical linear regression.

For the specific experiences with (non-)disclosure (research question 3), descriptive analyses were performed. As the variables were not normally distributed, Mann–Whitney U-tests were used to compare those with a general positive experience to those with a general negative experience. This provided insight into the factors explaining the general positive or negative experience with (non-)disclosure.

## Results

### Participant Characteristics

#### Response Rate

Of the larger study (N = 2000), after removing duplicates (caused by personnel going on multiple deployments) and personnel who had left active service, a total of N = 1627 eligible respondents were left. Of those, 63% (N = 1025) started the questionnaire, and 54% (N = 878) fully completed. Only completed questionnaires were used for further analyses. Compared to those who completed the questionnaire, those who did not were predominantly females (χ^2^(1, N = 1008) = 6.01, p = 0.014), more respondents had lower and middle education levels (χ^2^(2, N = 1008) = 7.25, p = 0.027), and consisted of more non-commissioned officers (χ^2^(2, N = 1006) = 8.26, p = 0.016). With incomplete questionnaires, the majority gave up while answering the mental health questions.

Of those who completed the questionnaire, 36.9% (N = 324) indicated having (had) MI and were included for the current study. One participant was not included for further analyses due to missing data on the sustainable employability measures, leaving a sample of N = 323 participants.

### Sample Characteristics

The sample characteristics can be found in Table 2. More detailed information on reported MI can be found in Online Appendix B. Comparing the sample characteristics to the characteristics of all Dutch military personnel, the following things should be noted: (1) the current sample had an overrepresentation of the army, air force and military police; and (2) the current sample included an over representation of older, higher educated and ranking military personnel.

**Table 2:** Sample characteristics military personnel with mental illness (N = 323)

	N	%
Demographics		
Sex		
Male	282	87.3
Female	41	12.7
Age		
< 21	0	0
21–30	27	8.4
31–40	106	32.8
41–50	95	29.4
51–60	89	27.6
> 60	6	1.9
Marital status		
Partner	251	77.7
Single	72	22.3
Educational level		
Low	30	9.3
Medium	175	54.2
High	118	36.5
Work related context		
Type of work		
Combat unit	162	50.2
Combat support unit	161	49.8
Military branch		
Marine	22	6.8
Army	166	51.4
Air-force	84	26.0
Military-police	20	6.2
Staff	30	9.3
Other	1	.3
Ranks		
Soldiers	15	4.6
Non-commissioned officers	173	53.6
Officers	135	41.8
Word demands		
Work demands (Range, M (SD))	1–5, 3.14 (.67)	
Mental health related variables		
Past or current mental illness		
Current mental illness	68	21.1
Past mental illness	255	78.9
Months since end mental illness (Median, M (SD))	10, 32.02 (52.57)	
Mental illness work related		
Yes	215	66.6
No	108	33.4
Severity of symptoms mental illness		
Severity (Range, M (SD))	1–10, 7.04 (2.01)	

#### Disclosure Decision and General Experience

Of the participants, 75.6% (N = 245) indicated having disclosed their MI to their supervisor.

#### Sustainable Employability and Well-Being at Work

The descriptives for the measures of sustainable employability and well-being at work can be found in Table 3.

**Table 3:** Descriptives measures of sustainable employability and well-being at work, separated for disclosers and non-disclosers

Measure	Disclosers (N = 245)	Non-disclosers (N = 78)	Total (N = 323)	Difference	
	M (SD)	M (SD)	M (SD)	Significance	Range
Work ability	7.65 (1.62)	8 (1.43)	7.73 (1.58)	n.s	1–10
Turn-over intention	2.04 (1.04)	2.15 (1.11)	2.07 (1.06)	n.s	1–5
Job satisfaction	3.84 (.94)	3.89 (.96)	3.85 (.94)	n.s	1–5
Burn-out	2.77 (.98)	2.66 (.89)	2.74 (.96)	n.s	1–5.78
Authenticity	5.65 (1.57)	5.66 (1.55)	5.65 (1.56)	n.s	1–7
Capability set for work	4.02 (2.27)	4.04 (2.15)	4.03 (2.24)	n.s	0–7

*n.s.* non-significant

### The Association Between the Disclosure Decision and the Measures of Sustainable Employability and Well-Being at Work

As can be seen from Table 4 (model 1), the disclosure decision (disclosure vs. non-disclose) was not significantly associated with any of the measures of sustainable employability and well-being at work, when controlling for demographics, work-context, and health variables.

**Table 4:** Hierarchical regression for past disclosure decisions (Model 1) and disclosure experiences (Model 2) and their association with sustainable employability and well-being at work

	Work ability		Turnover-intention		Job satisfaction		Burn-out		Autheniticy		Capability	
	Beta	95% CI	Beta	95% CI	OR	95% CI	Beta	95% CI	Beta	95% CI	Beta	95% CI
Model 1												
(Constant) (B)	7.88**	**6.69 to 9.08**	2.18 **	**1.33 to 3.03**	N/A	N/A	.96*	**.21 to 1.71**	5.15**	**3.88 to 6.43**	5.18**	**3.36 to 7.00**
Threshold = 0	N/A	N/A	N/A	N/A	.02**	**.00 to .09**	N/A	N/A	N/A	N/A	N/A	N/A
Threshold = 1	N/A	N/A	N/A	N/A	.08*	**.02 to .39**	N/A	N/A	N/A	N/A	N/A	N/A
Threshold = 2	N/A	N/A	N/A	N/A	.25	.05 to 1.23	N/A	N/A	N/A	N/A	N/A	N/A
Threshold = 3	N/A	N/A	N/A	N/A	2.96	.60 to 14.51	N/A	N/A	N/A	N/A	N/A	N/A
Demographics												
Gender female (vs. male)	.07	− .04 to .18	.12*	**.01** to **.24**	1.68	.88 to 3.24	− .08	− .19 to .03	.03	− .08 to .15	− .01	− .13 to .10
Age	.01	− .10 to − .11	− .18*	**− .29 to − .07**	.96	.77 to 1.19	− .06	− .17 to .05	− .07	− .18 to .05	− .05	− .16 to .06
Education	.06	− .05 to .17	− .08	− .19 to .03	.97	.67 to 1.39	− .03	− .14 to .08	.03	− .09 to .14	− .01	− .12 to .11
Work context												
Combat units (vs. combat support units)	.05	− .05 to .16	− .04	− .15 to .07	1.09	.71 to 1.68	− .05	− .16 to .06	.00	− .11 to .11	− .07	− .18 to .05
Work demands	− .14*	**− .24 to − .04**	− .03	− .14 to .08	1.34	.97 to 1.86	.14*	**.03 to .24**	.04	− .07 to .16	.06	− .05 to .17
Health context												
Mental illness current (vs. past)	− .33**	**− .44** to **− .23**	.16*	**.05 to .27**	.26**	**.15 to .43**	.22**	**.12 to .33**	**− .18**	**− .29 to − .07**	− .22**	**− .33 to − .11**
Symptom severity	− .10	− .21 to .01	.00	− .11 to .12	.95	.85 to 1.06	.20**	**.09 to .31**	− .08	− .19 to .04	− .10	− .21 to .02
Disclosure												
Decision (disclosure vs. non-disclosure)	− .05	− .16 to .05	− .04	− .15 to .08	.93	.55 to 1.56	− .02	− .13 to .09	.03	− .09 to .14	.02	− .10 to .13
Model summary												
R2	**.18**		**.08**		**.10**		**.13**		.05		**.06**	
Significance	F(8) = 8.69 **		F(8) = 3.39 *		**χ**^**2**^(8) = 31.57**		F(8) = 6.01**		F(8) = 1.88		F(8) = 2.39 *	
Model 2												
(Constant) (B)	7.31**	**6.02 to 8.60**	2.54**	**1.62 to 3.46**	N/A	N/A	1.49**	**.69 to 2.29**	4.09**	**2.73 to 5.44**	3.36*	**1.45 to 5.27**
Threshold = 0	N/A	N/A	N/A	N/A	.03**	**.01 to .19**	N/A	N/A	N/A	N/A	N/A	N/A
Threshold = 1	N/A	N/A	N/A	N/A	.15*	**.03 to .81**	N/A	N/A	N/A	N/A	N/A	N/A
Threshold = 2	N/A	N/A	N/A	N/A	.47	.09 to 2.58	N/A	N/A	N/A	N/A	N/A	N/A
Threshold = 3	N/A	N/A	N/A	N/A	5.64	1.01 to 31.42	N/A	N/A	N/A	N/A	N/A	N/A
Demographics												
Gender (female vs. male)	.06	− .04 to .17	.13*	**.02 to .24**	1.67	.87 to 3.22	− .07	− .18 to .04	.03	− .09 to .14	− .02	− .14 to .09
Age	.02	− .09 to .12	− .19*	**− .30 to − .08**	.98	.79 to 1.21	− .08	− .18 to .03	− .05	− .16 to .07	− .02	− .13 to .09
Education	.07	− .04 to .18	− .09	− .20 to .03	.99	.69 to 1.42	− .04	− .15 to .07	.04	− .07 to .16	.01	− .10 to .12
Work context												
Combat units (vs. combat support units)	.06	− .05 to .16	− .05	− .16 to .06	1.10	.72 to 1.70	− .06	− .17 to .05	.02	− .10 to .13	− .05	− .16 to .06
Work demands	− .14*	**− .25 to − .04**	− .03	− .14 to .08	1.32	.96 to 1.83	.15*	**.04 to .25**	.03	− .08 to .14	.05	− .06 to .15
Health context												
Mental illness current (vs. past)	− .33**	**− .43 to − .23**	.15*	**.04 to .26**	.26**	**.15 to .45**	.21**	**.11 to .31**	**− .17**	**− .28 to − .06**	− .20**	**− .31 to − .09**
Symptom severity	− .10	− .20 to .01	.00	− .11 to .11	.96	.85 to 1.07	.20**	**.09 to .30**	− .07	− .18 to .05	− .09	− .20 to .03
Disclosure												
Decision (disclosure vs. non-disclosure)	− .06	− .16 to .05	− .04	− .15 to .08	.91	.54 to 1.53	− .01	− .12 to .10	.02	− .10 to .13	.01	− .10 to .12
Experience (positive vs. negative)	.12*	**.02 to .22**	− .11*	**− .22 to − .00**	1.78	.97 to 3.28	− .18*	**− .28 to − .07**	**.22**	**.11 to .32**	.26**	**.15 to .36**
Model summary												
R2	**.19**		**.09**		**.11**		**.16**		**.09**		**.12**	
Significance	F(9) = 8.39 **		F(9) = 3.49 **		**(χ**^**2**^(9) = 34.97**		F(9) = 6.79**		F(9) = 3.50**		F(9) = 4.86 **	
R2 change	**.01**		**.01**		.01		**.03**		.05		**.07**	
Significance R2 change	F(1,313) = 5.08 *		F(1,313) = 3.99 *		(χ^2 difference^(1) = 3.40		F(1,313) = 11.36*		F(1,313) = 15.76**		F(1,313) = 23.22 **	

For job satisfaction an ordinal logistic regression was performed, as job satisfaction was not normally distributed

Bold indicates a significant effects, with *p < .05 and **p < .001

### The Association Between Disclosure Experience and Sustainable Employability and Well-Being at Work

As can be seen from Table 4 (model 2), disclosure experience (positive vs. negative) was significantly positively associated with almost all measures of sustainable employability and well-being at work, controlling for disclosure decision, demographics, work-context and health variables. Disclosure experience was not significantly associated with job satisfaction. Adding disclosure experience to the model, significantly improved the prediction of the model for all measures of sustainable employability and well-being at work, except for job satisfaction. Another important factor associated with sustainable employability and well-being at work, was whether the MI was current or in the past. Military personnel with current MI scored lower on all the measures of sustainable employability and well-being at work.

### General and Specific Experiences with the Disclosure Decision

A full overview of the disclosure experiences can be found in Table 1.

Of those who had *disclosed* (N = 245), 86.9% (N = 213) was (very) positive about their decision. The most often reported reasons for a positive experience were receiving support from a supervisor (90.1%) and the supervisor taking MI seriously (92.5%), indicating the important role of the supervisor. The most reported reasons for a negative experience were stigma related, namely experiencing gossip (81.3%), experiencing discrimination (78.1%), feeling shame (71.9%), and negative career consequences (65.6%). Additionally, the majority of those who had disclosed, in retrospect would have preferred to solve their own problems (84.4%). There was a significant difference between those with a general positive experience and those with a general negative experience for all these specific experiences. For example, those with positive experiences reported significantly more supervisor support (*M*_pos_ = 3.20, *M*_neg_ = 1.94, *p* < 0.001) compared to those who reported negative experiences with disclosure. Additionally, those with negative experiences reported significantly more shame discrimination (*M*_pos_ = 1.70, *M*_neg_ = 2.84, *p* < 0.001) and negative career consequences (*M*_pos_ = 1.69, *M*_neg_ = 2.56, *p* < 0.001) compared to those with positive experiences.

Of those who had not disclosed (N = 78), 84.6% (N = 66) was (very) positive about their decision. The most mentioned reasons for a positive experience were the preference to solve one’s own problems (self-management) (89.4%), MI remaining private (80.3%) and the MI not effecting occupational functioning (72.7%). The most mentioned reasons for a negative experience were that participants felt they had missed out on social support from their supervisor (75.0%) and that they had not been able to be their authentic selves (58.3%). Regarding the reported specific experiences, there were no significant differences in reported experiences for those with a general positive experience vs. those with a general negative experience. This is likely caused by the small sample size for those who did not disclose and had a negative experience (N = 12).

## Discussion

The current study examined the association between the disclosure decision (disclosure vs. non-disclosure of MI to a supervisor) and several measures of sustainable employability and well-being at work. The majority of military personnel with MI had disclosed their MI to their supervisor (75.6%). The current study found no significant association between this disclosure decision and any of the measures of sustainable employability and well-being at work. Although the current study did not find a direct association between disclosure decision and several measures of sustainable employability and well-being at work, the current study did show the importance of positive (non-)disclosure experiences. Disclosure experiences (positive vs. negative) were significantly related to almost all the measures of sustainable employability and well-being at work. The majority was positive about their disclosure decision (86.9%). Those with a positive disclosure experience reported significantly more support from their supervisors, showing the crucial role the supervisor had for positive disclosure experiences. Additionally, those with negative experiences with disclosure reported significantly more shame, discrimination, negative career consequences, and social rejection, indicating that stigma surrounding MI played a key role in negative disclosure experiences.

There was no significant association between disclosure decision and sustainable employability and well-being at work in the current study, possibly because positive and negative consequences of (non-)disclosure co-exist. Previous research suggests that there might be a negative association between disclosure of MI to a supervisor and sustainable employability. For example, a study among Dutch line managers showed that 64% was reluctant to hire someone with MI [17]. However, previous research has also suggested that there might be a positive association between disclosure of MI and sustainable employability. For example, disclosure can lead to opportunities for supervisor support and workplace accommodations [20–22]. A scoping review on workplace accommodations provided evidence that workplace accommodations are associated with longer job tenure [47]. It is likely that these positive and negative consequences of disclosure co-exist, and that therefore no direct association between disclosure decision and sustainable employability and well-being at work was found.

The current study included the disclosure experiences, and thus the consequences of disclosure, while most models on disclosure decision making focus on the antecedents of disclosure [28, 29]. A more complete model of disclosure, which does include consequences of disclosure in the work-context, is the *mental health condition disclosure decision-making process from antecedents to outcomes* model [31]. The original data supporting this model is of qualitative nature. The current study found that disclosure is associated with (1) alleviation of inhibition, as people could be their true and authentic self, (2) social support, (3) negative career consequences, (4) access to accommodations, (5) improved relationships, and (6) experiences of stigma. This is in line with what the model proposes, and there for the current study quantitively confirms this model.

Although no significant relationship between the disclosure decision and sustainable employability and well-being at work was found, the findings of the current study did show the importance of how the work environment (colleagues and supervisors) respond to disclosure. There was a significant association between positive disclosure experience and almost all measures of sustainable employability and well-being at work. This is in line with what the previously mentioned model proposes, namely that long term outcomes of disclosure (such as sustainable employability) are influenced by the short-term outcomes (disclosure experiences). It should however be noted that the model mostly focuses on outcomes of disclosure, while the current study also includes outcomes of non-disclosure. For example missed opportunities for support and work-accommodations, but also positive outcomes such as MI remaining private and being able to shield yourself from stigma and discrimination. Given the importance of positive disclosure experiences for sustainable employability and well-being at work, it is essential to examine what contributes to a positive or negative (non-)disclosure experience.

The findings indicate the key role the supervisor plays for a positive disclosure experience, and thus also for well-being at work and sustainable employability of workers with MI. The crucial role the supervisor plays in whether workers decide to disclose their MI has been shown before, both in military and civilian samples [24, 31]. A previous study in the Dutch military showed that the quality of the supervisor-employee relationship was significantly associated with the disclosure decision (manuscript submitted for publication). The current study now adds to the literature by showing the crucial role the supervisor also played in the consequences of disclosure, namely the disclosure experience. A previous study among Dutch workers in general also found that of those who were positive about disclosure, the majority indicated that they were supported by their supervisor [27]. This highlights the importance of the supervisor role both for disclosure decisions and experiences. These findings indicate the importance of providing training to help supervisors to improve understanding and support of MI needs [48]. The role of the supervisor is especially important in the military setting, due to the strong hierarchical structure in the military, making military personnel more dependent on their supervisors [49].

Stigma and discrimination also had a large impact on disclosure experiences. Those with negative experiences with disclosure were more likely to have experienced discrimination, negative career consequences, and social rejection. However, non-disclosure could not fully protect against stigma and discrimination either. Approximately half of those who did not disclose, still had experienced social rejection and negative career consequences, and had felt embarrassed. It is possible that people had disclosed MI to others in their environment, and that this caused social rejection and embarrassment. Previous research shows that fear of being stigmatized plays an important role in the disclosure decision itself, both in military and civilian samples [24, 27, 28, 33, 41]. The current study indicates that this fear is realistic, as those who disclosed, indeed experienced stigma and discrimination.

The experienced stigma and discrimination highlight the importance of destigmatizing interventions for well-being at work and sustainable employability. These interventions should target three groups: (1) *Supervisors:* As stigma and discrimination was frequently experienced by those who disclosed, interventions should target the supervisor to educate them about MI to take away possible stigma, and to help them to better support workers with MI [48]. (2) *Workers with MI:* the current study showed that both disclosure and non-disclosure can lead to stigma and discrimination. Previous research shows that the disclosure message and timing are important contributors for possible positive or negative outcomes of disclosure [22]. Therefore, to increase chances that disclosure will lead to work-adjustments, and to help workers to shield themselves from stigma and discrimination, disclosure decision aids could be used as they assist workers in making a well-considered disclosure decision. Additionally, support with disclosure decisions can reduce experienced self-stigma [50–52]. (3) *The public:* As those who did not disclose to their supervisor, still experienced stigma and discrimination, this suggests that the public-stigma surrounding MI should also be targeted. A meta-analysis on interventions targeting public-stigma showed that both education and contact between people with and without MI (in the right context, e.g. no power differences) have positive effects on reducing stigma [53].

### Strengths and Limitations

To the knowledge of the authors, this study was the first study to directly test the association between disclosure and sustainable employability and well-being at work. A strength of this study was that several measures of sustainable employability and well-being at work were used, combining traditional and novel measures. Additionally, the study included a large sample, and included participants that had not disclosed, a group that is usually hard to study.

However, there were also limitations which should be mentioned. First, the current study used a cross-sectional design, meaning that no conclusions about causality in the association between disclosure experiences and sustainable employability and well-being at work can be drawn. Outcome bias could partially explain that the majority was positive about their decision, and that positive experiences were associated with higher sustainable employability and well-being at work. Another disadvantage of the cross-sectional design used, is that sustainable employability and well-being at work was only assessed at one point in time, while ideally it should be assessed throughout the working life of a person [8]. Future research should study disclosure decision, disclosure experiences and sustainable employability and well-being at work longitudinally.

Second, an important limitation of the current study is that it only included active-duty military personnel. This means that those who had left the military, possibly after MI and disclosing, were not included in this study. Those with the most negative consequences of disclosure, such as loss of employment, were not represented in this study. Additionally, no data was available on time since disclosure, so no indication can be given about the time that military personnel have worked after their decision. Future research is needed to gain insight into this group, military personnel who have left active service.

Third, only a small sample of participants was included that did not disclose and were negative about this decision. This might explain the non-significant differences between those who are positive and negative about non-disclosure. Additionally, general (non-)disclosure experience was measured with a scale composing of two negative and two positive responses and no neutral response option. This forced participants to choose between a general positive or negative experience, while these experiences can co-occur. This might also explain the non-significant differences between those with a general positive and those with a general negative experience with non-disclosure.

Fourth, lower ranked and lower-educated military personnel were underrepresented in this study. Comparisons showed that lower ranking and lower educated personnel were less likely to have completed the questionnaire once started. Possibly, the questionnaire was hard to answer. Previous research has shown that younger and lower educated workers disclosed less [27], so disclosure rates in the current study might be an overestimation of the true rates.

Fifth, the current study did not include possibly important measures of individual differences such as personality traits and self-concept traits. These measures could also partly explain the results, as they can affect both the judgment about disclosure experience as the judgment about sustainable employability and well-being at work. For example, neuroticism could possibly be association with the disclosure decision [54], disclosure experiences and sustainable employability and well-being at work [55]. Future research should thus also include measures of individual differences, such as personality traits, to account for this.

## Conclusions

In our study, we found the actual disclosure decision (yes/no) not to be related to sustainable employability and well-being at work. However, we did find that how workers had experienced their disclosure decision was significantly associated with sustainable employability and well-being at work. It is important to increase the likelihood of positive disclosure experiences. Two important reasons for military personnel to be positive about their non-disclosure decision were that their MI remained private and that it prevented them from getting discriminated against. This highlights the importance of a safe work environment for disclosure, where non-disclosers also feel safe to disclose. Those who were positive about their disclosure decision, indicated that they had received support from their supervisor and work adjustments. When there is a safe work environment, more people will be inclined to disclose and can benefit from supervisor support and work adjustments. This can promote sustainable employability and well-being at work.

To create a work environment which is safe for disclosure, intervention studies should focus on 1) supervisor training on how to support workers with MI and 2) the stigma surrounding MI. Additionally, as research on the relationship between disclosure and sustainable employability and well-being at work is scarce, future research should further examine this relationship longitudinally.

## Supplementary Information

Below is the link to the electronic supplementary material.

Supplementary file1 (DOCX 15 kb)

Supplementary file2 (DOCX 14 kb)

Supplementary file3 (DOCX 27 kb)

## Data Availability

The data that support the findings of this study are available upon reasonable request from the corresponding author, R.I. Bogaers.
