# Supplementary material for: Workplace Mental Health Disclosure, Sustainable Employability and Well-Being at Work: A Cross-Sectional Study Among Military Personnel with Mental Illness
Source: J Occup Rehabil. 2022 Nov 14;33(2):399–413. doi: 10.1007/s10926-022-10083-2 (PMC9663181; doi:10.1007/s10926-022-10083-2)
Supplement: Supplementary file 1 — Supplementary file1 (DOCX 15 kb) [file 10926_2022_10083_MOESM1_ESM.docx]

| **Appendix A. Measures used to assess current mental illness and substance abuse.** | | | | | |
| --- | --- | --- | --- | --- | --- |
| **Scale** | **Information** | **Psychometric properties based on earlier studies** | **Reliability in current study** | **Cut-off score used** | **References** |
| The hospital anxiety and depression scale (HADS). | A 14-item scale measuring anxiety and depression. | Based on a literature review, reliability of the anxiety scale of the HADS varies from .68 to .93 and the depression scale varies from .67 to .90. Sensitivity and specificity for both anxiety and depression was approximately .80. | Depression: α=.85  Anxiety: α=.84 | A cut-off score of > 8 was used for depression and anxiety, as recommended by earlier research. | [31] |
| PTSD checklist for the DSM-5 (PCL-5) | 20-item scale measuring PTSD symptoms. Participants received a question screening whether they had experienced extremely stressful events (examples were provided), and if yes, they received the PCL-5. | The PCL-5 has strong reliability (α = .94) and convergent (rs = .74 to .85) and discriminant (rs=.31 to .60) validity. Sensitivity is approximately .77 and specificity .96. | α =.94 | A cut-off of >33 was used as an indication of PTSD, following the guidelines. | [34] |
| ASSIST-LITE | Measure to assess a wide range of substance (ab)use. This questionnaire consists of 6 items, one per substance, and 2-3 follow up questions in case a substance is used by the participant in the past 3 months. | Sensitivity for each substance was between .8 and 1.0, and specificity between .7 and .8. Reliability is N.A. as all questions are about different substances. | N/A as all questions are about different substances. | A cut-off of ≥ 2 was used for all substances except for alcohol, where the cut-off was ≥ 3, following the user manual. | [32] |
| AUDIT-C | A 3-item scale, to assess alcohol abuse. | The AUDIT-C has been validated and shown to have good internal consistency in a variety of different samples. In a previous study among military personnel, the reliability was .77. Specificity is between .89 and .91 and sensitivity between .73 and .86. | α=.64 | A cut-off of ≥ 8 was used, as recommended for military population. | [33] |
