# Supplementary material for: Workplace Mental Health Disclosure, Sustainable Employability and Well-Being at Work: A Cross-Sectional Study Among Military Personnel with Mental Illness
Source: J Occup Rehabil. 2022 Nov 14;33(2):399–413. doi: 10.1007/s10926-022-10083-2 (PMC9663181; doi:10.1007/s10926-022-10083-2)
Supplement: Supplementary file 2 — Supplementary file2 (DOCX 14 kb) [file 10926_2022_10083_MOESM2_ESM.docx]

| **Appendix B. Mental illness and substance abuse scores.** | | |
| --- | --- | --- |
|  | **Military personnel who indicated having (had) MI (N=324)** | |
|  | **N** | **%** |
| **Type of mental illness as reported by military personnel themselves** | | |
| Anxiety (incl. obsessive compulsive disorder) | 111 | 34.3 |
| Depression (incl. manic and bipolar) | 146 | 45.1 |
| Burn-out | 176 | 54.3 |
| Stress | 260 | 80.3 |
| Exhaustion | 205 | 63.3 |
| Post traumatic stress disorder | 55 | 17.0 |
| Psychotic disorders | 3 | .9 |
| Personality disorder | 77 | 23.8 |
| Autism | 16 | 5.0 |
| Attention deficit hyperactivity disorder | 34 | 10.5 |
| Eating disorder | 10 | 3.1 |
| Substance abuse | 56 | 17.3 |
| **Current type of mental illness based on measures of mental health** | | |
| HADS_depression | 59 | 18.2 |
| HADS_anxiety | 65 | 20.1 |
| Assist_lite_tobacco | 48 | 14.8 |
| Assist_lite_alcohol | 50 | 15.4 |
| Assist_lite_cannabis | 1 | .3 |
| Assist_lite_amphetamine | 2 | .6 |
| Assist_lite_sleepmedication | 8 | 2.5 |
| Assist_lite_streetdrug | 0 | 0.0 |
| Audit_C | 13 | 4.0 |
| PCL-5 score | 19 | 5.9 |
