# Supplementary material for: Workplace Mental Health Disclosure, Sustainable Employability and Well-Being at Work: A Cross-Sectional Study Among Military Personnel with Mental Illness
Source: J Occup Rehabil. 2022 Nov 14;33(2):399–413. doi: 10.1007/s10926-022-10083-2 (PMC9663181; doi:10.1007/s10926-022-10083-2)
Supplement: Supplementary file 3 — Supplementary file3 (DOCX 27 kb) [file 10926_2022_10083_MOESM3_ESM.docx]

STROBE Statement—checklist of items that should be included in reports of observational studies

| **Page** | | **Page** | |
| --- | --- | --- | --- |
| **No. Recommendation No.** | | | |
| **Title and abstract** | 1 | (*a*) Indicate the study’s design with a commonly used term in the title or the abstract | 1-3 |
|  |  | (*b*) Provide in the abstract an informative and balanced summary of what was done and what was found | 3 |
| **Introduction** |  |  |  |
| Background/rationale | 3-4 | Explain the scientific background and rationale for the investigation being reported | 4 |
| Objectives | 4-5 | State specific objectives, including any prespecified hypotheses | 4-5 |
| **Methods** |  |  |  |
| Study design | 5 | Present key elements of study design early in the paper | 5 |
| Setting | 5 | Describe the setting, locations, and relevant dates, including periods of recruitment, exposure, follow-up, and data | 5-6 |
|  |  | collection |  |
| Participants | 5 | *Cross-sectional study*—Give the eligibility criteria, and the sources and methods of selection of participants | 5-6 |
| Variables | 5-7 | Clearly define all outcomes, exposures, predictors, potential confounders, and effect modifiers. Give diagnostic criteria, if applicable | 6-9 |
| Data sources/ measurement | 5-7 | For each variable of interest, give sources of data and details of methods of assessment (measurement). Describe comparability of assessment methods if there is more than one group | 6-8 |
| Bias | N/A | Describe any efforts to address potential sources of bias | 15-16 |
| Study size | 8-9 | Explain how the study size was arrived at | 5-6 & 9 |
|  |  |  |  |
| Quantitative variables | 8 | Explain how quantitative variables were handled in the analyses. If applicable, describe which groupings were chosen and why | 8-9 |
| Statistical methods | 8 (*a*) | Describe all statistical methods, including those used to control for confounding | 8-9 |
|  |  | (*b*) Describe any methods used to examine subgroups and interactions | 8-9 |
|  |  | (*c*) Explain how missing data were addressed | 9 |
|  |  | (*d*) *Cross-sectional study*—If applicable, describe analytical methods taking account of sampling strategy | N/A |
|  |  | (*e*) Describe any sensitivity analyses | N/A |
| **Results** |  |  |  |

| Participants | 8-10* | (a) Report numbers of individuals at each stage of study—eg numbers potentially eligible,  examined for eligibility, confirmed eligible, included in the study, completing follow-up, and analysed | 9-10 |  |
| --- | --- | --- | --- | --- |
|  |  | (b) Give reasons for non-participation at each stage | 9-10 |  |
|  |  | (c) Consider use of a flow diagram | N/A |  |
| Descriptive data | 9-10* | (a) Give characteristics of study participants (eg demographic, clinical, social) and information on | 10 |  |
|  |  | exposures and potential confounders |  |  |
|  |  | (b) Indicate number of participants with missing data for each variable of interest | 9 |  |
|  |  | *Cross-sectional study—*Report numbers of outcome events or summary measures | 10 |  |
| Main results | 9-14 | (*a*) Give unadjusted estimates and, if applicable, confounder-adjusted estimates and their precision (eg, 95% confidence interval). Make clear which confounders were adjusted for and | 12-13 |  |
|  |  | why they were included |  |  |
|  |  | (*b*) Report category boundaries when continuous variables were categorized | 8-9 |  |
|  |  | (*c*) If relevant, consider translating estimates of relative risk into absolute risk for a meaningful | N/A |  |
|  |  | time period |  |  |

| Other analyses | 8 Report other analyses done—eg analyses of subgroups and interactions, and sensitivity analyses | 10-13 |  |
| --- | --- | --- | --- |
| **Discussion** |  |  |  |
| Key results | 14 Summarise key results with reference to study objectives | 13 |  |
| Limitations | 17-18 Discuss limitations of the study, taking into account sources of potential bias or imprecision. Discuss both direction and magnitude of any potential bias | 15-16 |  |
| Interpretation | 18 Give a cautious overall interpretation of results considering objectives, limitations, multiplicity of analyses, results from similar studies, and other relevant evidence | 13-17 |  |
| Generalisability | 17-18 Discuss the generalisability (external validity) of the study results | 15-16 |  |
| **Other information** |  |  |  |
| Funding | 21 Give the source of funding and the role of the funders for the present study and, if applicable, for the original study on which the present article is based | 23 |  |
